# QuickStats: Percentage*^,^^†^ of Adults Aged ≥20 Years Who Consumed Fruit on a Given Day, by Race and Hispanic Origin^§^ — United States, 2015–2018

**DOI:** 10.15585/mmwr.mm7036a5

**Published:** 2021-09-10

**Figure:**
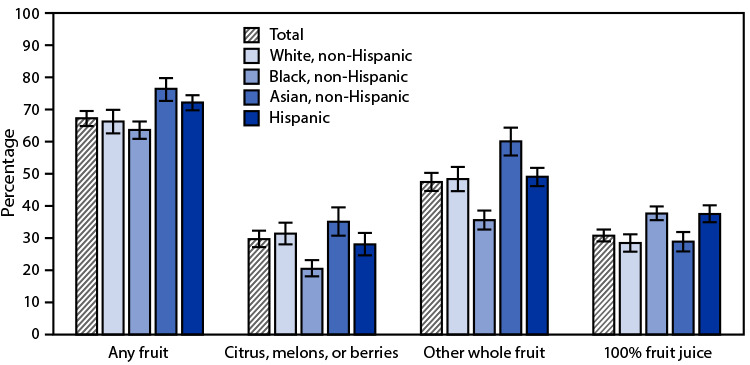
During 2015–2018, on a given day, 67.3% of adults aged ≥20 years consumed any fruit; 29.7% consumed citrus, melons, or berries; 47.5% consumed other whole fruits; and 30.8% consumed 100% fruit juice. Non-Hispanic Asian (76.5%) and Hispanic adults (72.2%) were more likely to consume any fruit on a given day than non-Hispanic White (66.3%) and non-Hispanic Black adults (63.7%). Non-Hispanic Black adults were least likely to consume citrus, melons, or berries (20.5%) and other whole fruit (35.6%), and non-Hispanic Asian adults were most likely to consume other whole fruits (60.1%). A higher percentage of non-Hispanic Black (37.7%) and Hispanic (37.5%) adults consumed 100% fruit juice compared with non-Hispanic White (28.5%) and non-Hispanic Asian (28.9%) adults.

**Source:** NCHS Data Brief, no. 397, National Center for Health Statistics. https://www.cdc.gov/nchs/data/databriefs/db397-H.pdf

